# Urinary tract infection associated with indwelling catheters in a intensive care unit: a preventive approach

**DOI:** 10.1186/2197-425X-3-S1-A1019

**Published:** 2015-10-01

**Authors:** MR Silva, MDCF Araujo, THRC Almeida, EC Santos, T Figueiredo, DA Barbosa

**Affiliations:** Universidade Estadual de Santa Cruz, Health Sciences, Ilhéus, Brazil; Universidade Federal de São Paulo, Health Sciences, São Paulo, Brazil; Universidade Estadual de Santa Cruz, Health Sciences, Ilheus, Brazil; Base Hospital Luis Eduardo Magalhães, Nursing, Itabuna, Brazil

## Introduction

Urinary tract infection when associated with bladder catheterization delay is one of the most common causes of infection related to health care, accounting for 40% of the incidence. Much of microorganisms that contaminate the drainage system stems from inadequate aseptic technique and hygiene oversights in the microbiota of the perineal and perianal region. It is an infection with high preventive potential, suggesting the need for educational interventions, with the purpose to train professionals, aimed at prevention and health promotion.

## Objective

To evaluate the incidence after educational intervention on bladder catheterization delay on the insertion technique and maintenance care, the nursing staff.

## Methodology

Descriptive, exploratory, quantitative study in a hospital in the southern region of Bahia-Brazil. Data collection was conducted from March to November 2014. The study population were 50 patients admitted to the intensive care unit. The Ethics Committee protocol and the Federal University of São Paulo: 319 255/2013.

## Results

Prevalence of infections in male patients: 35 (70%). Diagnostic: Encephalopathy 16 (32%). Age group: 68-87 years old: 23 (46%). Length of stay: 1 to 17 days: 43 (86%). Type catheter material Foley and Latex: 50 (100%). Catheter dwell time: 6 to 10 days: 23 (46%). Laboratory tests confirmed: 12 (24%), and the Escherichia coli frequently identified microorganism. No infection: 38 (76%). The indicators assessed as adequate in 100%: indication of the catheter, closed drainage system maintenance, positioning the collection bag, clear urine flow. In 32 (64%): Fixing the catheter, still requires greater vigilance.

## Conclusion

After training and constant supervision of professionals and adherence to measures to prevent and control the catheter, it was possible to identify the profile of the unit and realize a reduction in the incidence of urinary tract infection.

## Grant Acknowledgment

Follow the recommendations of best practices to provide direct patient care, the teacher serves as a model and operates in favor of its security, the teams and patients. Pay attention to the infection indicators is quality of care provided to patients.
